# Toxic metals and metalloids in collagen supplements of fish and jellyfish origin: Risk assessment for daily intake

**DOI:** 10.1515/med-2025-1141

**Published:** 2025-04-15

**Authors:** Gaetano Cammilleri, Marina Tortorici, Licia Pantano, Francesco Giuseppe Galluzzo, Andrea Pulvirenti, Maria Drussilla Buscemi, Gianluigi Maria Lo Dico, Andrea Macaluso, Vittorio Calabrese, Ursula M. Jacob, Vincenzo Ferrantelli

**Affiliations:** Experimental Zooprophylactic Institute of Sicily, 90129 Palermo, Italy; Department of Life Sciences, University of Modena and Reggio Emilia, 41121 Modena, Italy; Department of Biomedical and Biotechnological Sciences, University of Catania, 95123 Catania, Italy; Healthcare AG, 8001 Zürich, Switzerland; Experimental Zooprophylactic Institute of Sicily, Via Gino Marinuzzi 3, 90129 Palermo, Italy

**Keywords:** marine collagen, contaminants, ICP-MS, toxicology

## Abstract

**Aim:**

We examined marine collagen supplements derived from fish and jellyfish for the presence of toxic metals and metalloids (Pb, Cd, Cr, Hg, and As). A risk assessment was also carried out by converting the obtained concentrations into average daily doses (ADDs) and comparing them with tolerable daily intakes (TDIs) to evaluate potential health risks associated with long-term consumption.

**Methods:**

The levels of Pb, Cd, Cr, and As in marine collagen samples were quantified using inductively coupled plasma mass spectrometry (ICP-MS). Mercury levels were analysed with a direct analyser. The study analysed various brands of marine collagen supplements from fish and jellyfish, ensuring compliance with EU regulatory limits.

**Results:**

Arsenic was the most abundant element detected, with mean values of 0.59 ± 0.28 mg/kg, followed by Pb (0.13 ± 0.02 mg/kg). Significant variability was observed between brands. Hg was detectable in only 12% of the samples. Marine collagen samples derived from jellyfish and *Scomber scombrus* skin extract showed no detectable toxic metals or metalloids. None of the samples exceeded EU regulatory limits, and ADDs were consistently below TDIs, indicating safety for consumption at recommended doses. However, it is recommended that food safety regulations be updated to account for potential cumulative risks from simultaneous intake of contaminated supplements.

## 1 Introduction

The appearance and integrity of the skin can be compromised by factors such as photoaging, nutritional deficiencies, hormonal disorders, and environmental factors [1].

The skin consists of two layers: the outer layer, called the epidermis, and the inner layer, known as the dermis. The epidermis, being non-vascular, depends on the dermis for nutrient supply. This process gradually slows down with age, leading to a decline in the quality of collagen.

Collagen is a group of proteins that occurs naturally. Their proteins are so long, with a fibrous structure that has different functions (from globulars to enzyme) [2]. Collagen is one of the most abundant groups of protein in invertebrates and vertebrates (up to 25% of total protein content in many animals) [3] and represents the main protein of the connective tissue as well as a major component of the extracellular matrix. Their fibres support most tissues and structure the cells from the outsides. Commonly, the amino acid sequence of collagen is composed of glycine–proline–X and glycine–X–hydroxyproline, where X can be another glycine and proline or hydroxyproline [4].

Studies have shown that the synthesis of this protein varies in different phases of life. It is estimated that the natural reintegration capacity with the body decreases about 1.5% per year. Therefore, there is an endogenous production that progressively loses its effectiveness over time, which could be compensated by external uptake [5]. The aging process also involves the loss of hyaluronic acid. At the same time, the density of collagen and elastin in the dermis decreases, resulting in the deterioration of the structure and elasticity of the skin. This gradual decrease is induced not only by genetic variations but also by extrinsic components (reactive oxygen species [ROS], primarily produced by UV radiation and cellular metabolism) that lead to structural changes affecting skin cells and dermal tissue structure [5].

In recent years, there was an increasing attention on the skin care that led to an increase of collagen and collagen-based products. Collagen peptides by oral uptake, stimulate the production of hyaluronic acid in dermal fibroblasts and improve skin barrier function in addition to stimulating endogenous collagen production [6]. For these reasons, collagen has anti-wrinkling and anti-aging properties even by protecting against UV radiation [7].

The literature has shown the effectiveness of collagen in improving skin parameters such as trans epidermal water loss, elasticity, and reduction of wrinkles [6].

Collagen can be obtained from various organisms. In the last few decades, collagen was preferentially extracted from terrestrial animals such as bovine skin, bone, and tendon as well as pig skin. However, the risks of the bovine spongiform encephalopathy, transmissible spongiform encephalopathy, and swine influenza from animals to humans were the main reasons that reconsidered these animals as source for collagen production [3,8]. One alternative is the collagen from marine origin which consists of collagen extracted from jellyfish, sponges and fish bones, skin, scales, and fins.

Considering this, at present marine organisms are the most used sources for collagen production in cosmetology because of their unique characteristics, such as the high bioavailability [9], the low environmental impact due to the use of fish processing industries waste [10], and the lower denaturation temperature. Therefore, in the future, marine collagen could definitively replace the biomaterial traditionally obtained from cattle and pigs, in which the commerce on an annual basis is up to 300,000 tons.

Most of the fish used for collagen production are Osteichthyes that occupy a high position in the marine food web, showing a high tendency to accumulate xenobiotics such as heavy metals and metalloids [11–15]. Owing to anthropic activities such as refining and electroplating, these elements enter into the aquatic ecosystem and bioaccumulate in these organisms [16]. The bioaccumulation can be represented by a combination of bioconcentration and absorption of xenobiotic compounds through the nutrition [17]. Many toxic elements such as chromium (Cr), cadmium (Cd), lead (Pb), and arsenic (As) were found in aquatic organisms and transferred into food products and supplements with hazardous concentrations [18–20].

According to the report from the International Agency for Research on Cancer (IARC) in 2018, hexavalent Cr has been designated as a Group I occupational carcinogen [21]. High levels of Cr induce oxidative stress and the production of ROS, targeting the DNA and lipid content of cells. This, in turn, results in DNA damage and lipid peroxidation, respectively.

Exposure to Cd may lead to painful degenerative bone disease, kidney, and lung diseases. Osteoporosis and bone fractures could be observed as a result of Cd toxicity [21]. Oppositely, exposure to Pb can lead to neurological, respiratory, urinary, and cardiovascular disorders, primarily through mechanisms involving immune modulation, oxidative stress, and inflammation. In the case of As, both acute and chronic toxicity are linked to the dysfunctions of numerous vital enzymes, similar to the effects observed with heavy metals.

At present, metal intoxication arisen from supplements has been an issue of great concern [22].

It is therefore essential to reduce the concentrations of these toxic elements from marine collagens. If this issue is not overcome, the use of marine collagen in food supplements production could be limited or regulatory bodies must pay more attention to the marine species used in the preparation of these supplements.

Most of the collagen production companies provide poor information on the type of fish and parts used for the production. In fact, producers can probably also use organs with high toxic metal storage ability.

As mentioned before, the chronic exposure to toxic metals can lead to their accumulation in vital organs such as liver, kidneys, heart, and brain, affecting their normal function. Such dysfunctions can lead to immunotoxicity, reproductive deficits, teratogenicity, endocrine toxicity, and carcinogenic effects [23,24].

As far as we know, few data on toxic metals in collagen supplements from marine origin are available, limiting a reliable risk assessment on their intake.

This study aimed at verifying the possible presence of As, Cr, Cd, and Pb in collagen supplements of fish origin by a validated inductively coupled plasma mass spectrometry (ICP-MS) method, in order to provide toxicological data on this type of products. The detection of Hg was also carried out by the use of a direct mercury analyser based on atomic absorption spectroscopy.

A risk assessment was also carried out to verify the possible chronic effects following their consumption.

## 2 Methods

### 2.1 Reagents and gases

ICP-MS grade water was obtained by Milli-Q^®^ purification system (Millipore, Bedford, MA, USA). The calibration solutions for the elements considered were obtained from VWR International LTD (Randon, Pennsylvania, USA). All the calibration solutions are of analytical grade. A tuning solution for ICP-MS containing Ce, Co, Li, Mg, Tl, and Y at 1 µg/L concentration was acquired from Agilent Technologies (Santa Monica, CA, USA). Ultra-pure nitric acid (60% V/V) was acquired from Merck KgaA (Darmstadt, Germany). Acetic acid ≥99% and NaOH 97% were purchased from Sigma Aldrich (Amsterdam, Netherlands). Ultra-pure carrier gas (Ar, 99.9995% pure) and dilution gases (He, 99.9995% pure; H2, 99.9995% pure) were acquired from SOL S.P.A. (Monza, Italy).

### 2.2 Sample collection

Collagen supplements of fish origin were purchased on e-commerce platforms and large-scale retail trade during 2022 and 2023. We excluded from the analysis the supplements which do not report on the label the species used for the extraction of the collagen. Of the 65 brands examined, only 4 brands reported this information on the label; therefore, 6 samples from 5 different production batch (i.e., products made in the same manufacturing run) were collected for each brand, for a total of 120 samples. The collagen extracted by the brands examined was obtained from Atlantic Cod (*Gadus morhua*; brand 1), Pangasius (*Pangasianodon hypophthalmus*; brand 2), Jellyfish (*Rhizostoma pulmo*; brand 3), and Tilapia (*Tilapia* spp. Brand 4). Furthermore, collagen was extracted from the skin of *Scomber scombrus* samples according to the protocol of Wijaya et al. [25] based on the use of 0.5 M acetic acid (*S. scombrus* collagen extract). Before the extraction, the *S. scombrus* samples were analysed by visual inspection to verify the integrity of the organs and the presence of visible nematode parasites of the Anisakidae family that could have collagenolytic activity (Malagón et al. [26]).

### 2.3 Sample extraction and trace elements determination

The samples were subjected to digestion following previously documented protocols, albeit with adjustments made to the initial weight [19,27,28]. About 0.1 ± 0.001 g of the collagen supplement samples was weighed and put on PTFE vessels with 3 mL of 60% (V/V) ultrapure nitric acid and 5 mL of ultrapure water. The samples were mineralized on an UltraWAVE digestion system (Milestone, Sorisole, Italy). Subsequently, ultrapure water was added to the samples up to 50 mL of volume. After a filtration with 0.45 µm filters, the samples were subjected to ICP-MS analysis. A 7700x series ICP-MS (Agilent Technologies, Santa Monica, CA, USA) was used for the detection of As, Cr, Cd, and Pb with the instrumental conditions reported in Table 1.

**Table 1:** Inductively coupled plasma mass spectrometry instrumental conditions used

Parameter		Value
Carrier gas		1.0 mL min^−1^
Reflect power		<30
Plasma gas flow		15 L min^−1^
Auxiliary gas flow		0.9 mL min^−1^
Spray chamber temperature		+2°C
Lens voltage		6.25 V
Mass resolution		0.7

Daily optimization of these parameters was conducted using a tuning solution to amplify the signal and reduce interference effects from polyatomic ions and doubly-charged ions. The acceptance criteria for resolution included maintaining relative standard deviation values below 3%.

The analysis was carried out using calibration curves, with 7 points equivalent to the standards and the calibration blank, admitting a correlation coefficient *r* ^2^ > 0.999. The analytical batch included the examination of Certified Reference Material DORM-5 (fish protein). The obtained values verified the precision and accuracy of the analytical determination. Calibration, precision, and trueness by recovery for the ICP-MS method were calculated according to European and international regulations [29,30]. The recovery of the method was calculated considering three concentration points in order to evaluate its trueness with acceptance limits ranging from 90 to 110%. All the samples were spiked with the right amount of element concentration and digested according to the established microwave programmes, and then analysed. The recovery calculation was carried out using the formula:

(1) \[\text{Recovery}\hspace{.25em}( \% )=100\hspace{.25em}C/\text{spiked}\hspace{.25em}\text{concentration}]\]

where *C* is the concentration of the element. The repeatability and within-laboratory reproducibility were assessed using the percentage variation coefficient (CV%) of the samples used for the recovery study over three different days. Regarding the within-laboratory reproducibility, the analysis of the first day was done with no changes; in the second and third days, operators were changed. The limit of detection (LOD) was assessed by the 3σ approach, while the limit of quantification (LOQ) was calculated with the 10σ approach [31].

The Hg determination was carried out using a DMA-80 analyser (Milestone, Wesleyan University, Middletown, CT, USA). A fixed wavelength of 250 nm was chosen for the Hg determination using atomic absorption spectroscopy.

Calibration curves with ≥98% analytical grade standard and determination of certified reference materials (Fapas) with duplicate samples were carried out for the quality assessment.

The calibration curve was generated by correlating a value of absorbance with each known mercury concentration, encompassing five concentration points ranging from 0.050 to 2 mg/kg. The instrumentation was set up with the following parameters: combustion temperature: 650°C; catalyst temperature: 565°C; cuvette temperature: 125°C; decomposition temperature: >600°C; start max temperature: 250°C; purge time: 60 s; and time for signal registration: 30 s.

### 2.4 Data collection and statistical analysis

The data obtained were expressed as mg/kg wet weight (w.w.). Not detected elements were considered as half of the LOQ for the statistical analysis [22]. The data groups were subjected to Shapiro–Wilk analysis to test the normality of distribution. A Kruskal–Wallis test, using the R 4.1.2. software, was used to verify significant differences between collagen supplements brands.

### 2.5 Health risk assessment

The average daily intake (ADDs), expressed per kg bodyweight (µg/kg/day) was calculated to estimate the risk of a potentially harmful intake for all the elements examined. It was assumed that either 5 or 10 g of supplements are consumed daily, based on the recommended doses specified on the label. The calculations were carried out as follows:

(2) \[\text{ADD}=(\text{DFC}\times C)/\text{BW}]\]

where *C* is the concentration of the element (As, Cr, Cd, and Pb) in the sample analysed (mg/kg), DFC is the daily intake, and BW is the average body weight. As confirmed previously [32,33], women use this type of supplements to a greater extent than man therefore, the calculations were carried out considering women with normal weight (67.7 kg [22]). Normal weights were represented by arithmetic means reported by Filipsson et al. [34].

The ADD was compared with the tolerable daily intake (TDI) of all the elements analysed reported in literature (Table 2) [22]. The risk assessment for As was conducted, considering the assumption set forth by the European Food Safety Authority for inorganic arsenic (iAs) [35].

**Table 2:** TDIs of the elements examined, along with the critical endpoints considered for TDI determination

Element	TDI and critical endpoint
As	0.3–8 μg/kg/day according to BMDL01 interval presented by EFSA for iAs. The critical endpoints are cancers of the lung and bladder and dermal lesions [36]
Cd	0.36 μg/kg/day. The EFSA tolerable weekly intake (2.5 μg/kg/week) was set based on the critical endpoint for kidney toxicity [37]
Pb	0.5 μg/kg/day. The lowest BMDL01 level after oral Pb exposure is 0.5 μg/kg/day. Critical endpoint: reduction in renal function assessed through the glomerular filtration rate [38]
Cr	0.3 µg/kg/day. EFSA established a BMDL01 of 0.17 mg/kg/day. Critical end point: renal, hepatic, or gastrointestinal toxicity for oral exposure [39]


**Ethical approval:** The collagen supplements analysed was purchased from the e-commerce. No animal experiment was conducted. No experiments on humans were done during the study.

## 3 Results and discussion

**Figure 1:**
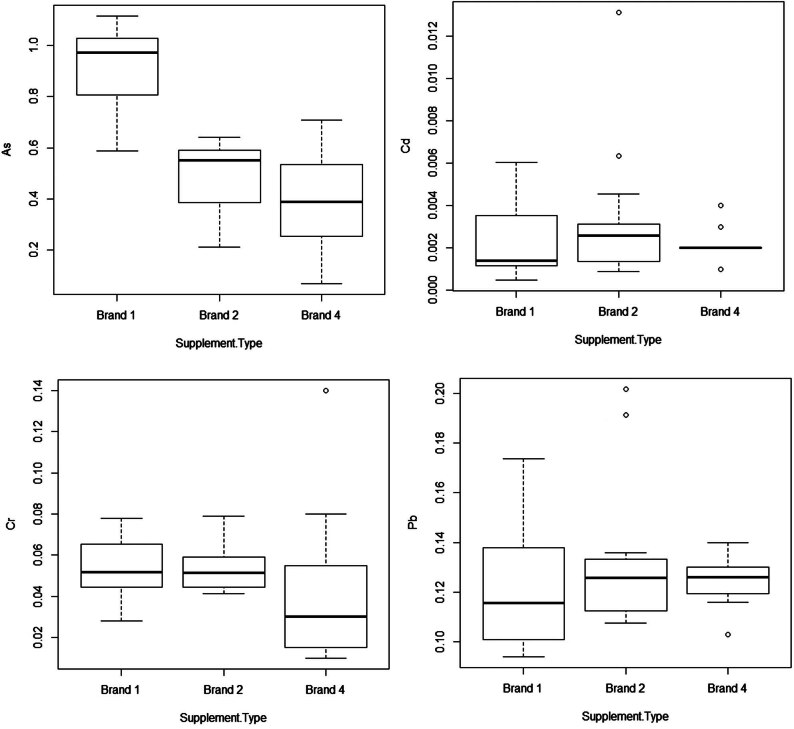
Box whisker plots of As, Cd, Cr, and Pb concentration in the collagen supplements examined. Circles represent the outliers.

The linearity tests for As, Cr, Cd, Hg, and Pb showed *r* ^2^ values greater than 0.999. The LODs and LOQs of the method were in accordance with the European Union regulations (333/2007 EC; 2002/657 Commission Decision). Satisfactory recovery values were obtained for all the elements examined (96–105%). The proposed method confirmed ICP-MS as the favourite technique for the rapid multi-elemental analysis of food matrices. It provides a unique combination of multi-elemental capability, high sensitivity, and selectivity, all within a short timeframe (only 2 min per sample). ICP-MS, in contrast to atomic absorption techniques, offers higher speed, precision, and sensitivity, while also minimizing matrix interference. Additionally, the technique uses very small quantities, leading to a significant reduction in terms of cost of sample analysis compared to alternative methods. The mean contents of As, Cr, Cd, and Pb are shown in Table 3, while the data distributions are shown in Figure 1.

**Table 3:** Toxic metal concentrations of the marine collagen samples examined expressed as mg/kg

	As			Cr			Cd			Pb		
	Mean ± SD	Min	Max	Mean ± SD	Min	Max	Mean ± SD	Min	Max	Mean ± SD	Min	Max
Brand 1	0.912 ± 0.16	0.582	1.11	0.054 ± 0.02	0.03	0.08	0.002 ± 0.002	0.001	0.006	0.123 ± 0.023	n.d.	0.038
Brand 2	0.484 ± 0.16	0.212	0.64	0.053 ± 0.01	0.04	0.08	0.003 ± 0.003	0.001	0.013	0.136 ± 0.03	0.107	0.201
Brand 3	n.d.	—	—	n.d.	—	—	n.d.	—	—	n.d.	—	—
Brand 4	0.390 ± 0.2	0.1	0.71	0.040 ± 0.03	0.01	0.14	0.002 ± 0.001	0.001	0.011	0.125 ± 0.01	0.103	0.14
*S. scombrus* collagen extract	n.d.	—	—	n.d.	—	—	n.d.	—	—	n.d.	—	—
Total	0.591 ± 0.28	0.1	1.11	0.050 ± 0.01	0.028	0.079	0.003 ± 0.002	0.0007	0.013	0.138 ± 0.02	0.094	0.201

n.d. = not detected.

No toxic metals and metalloid contents were found in all the marine collagen samples from jellyfish and from *S. scombrus* skin. Regarding the marine collagen of fish origin, trace contents of Cr, As, and Pb were found in all the samples examined. Cd was found in 98% of the samples analysed; only three samples showed a Cd content below the LOD of the method. Conversely, mercury was found in only two samples from brand 2, showing a maximum of 0.0018 mg/kg.

The mean levels of the toxic metals and metalloids examined followed the order As > Pb > Cr > Cd > Hg.

As was the most abundant trace element, with a maximum concentration of 1.11 mg/kg in a marine collagen sample of brand 1. The mean concentrations of As in brand 1 were significantly higher than the other brands (*p*-value = 3 × 10^−7^). As is a prevalent environmental metalloid that has been classified as a Group 1 human carcinogen by the IARC. As exerts its toxic effects on various tissues by binding to the sulfhydryl groups of proteins. Both acute and chronic exposure to As are associated with non-cancer health effects as well as various types of cancer, including skin, bladder, liver, and kidney cancer [40–42]. Conversely, similar average Cr and Cd levels were found in the three brands analysed, showing maximum values in the samples from Pangasius (brand 2; 0.08 and 0.01 mg/kg, respectively). Regarding Pb, brand 2 showed a higher number of samples to the upper quartile of the data distribution.

The Pb, Cd, and Cr levels found in this work were much lower than what was found by Cutajar et al. [9] in collagen extracts from Atlantic Bluefin Tuna discards, probably due to the fish species employed for the collagen extraction. Adult bluefin tuna feeding at a high trophic position determining high bioaccumulation of xenobiotics such as toxic metals due to biomagnification phenomena [43].

Higher mean Cr, Pb, and Cd values were found in the marine collagen samples derived from Pangasius (*P. hypophthalmus*) (brand 2); the data distributions of Pb, Cr, and Cd of the samples of this brand are negatively skewed, showing a high number of samples toward the upper end of the min–max concentration span. This difference could be due to the extraction procedure used and the type of fish used for the collagen production, as confirmed by the results on *S. scombrus* collagen samples from skin, which revealed no detectable trace metals and metalloid contents, in accordance with the low heavy metal contents found in bibliography for this species [44]. Pangasius is a suitable bioaccumulator of toxic metals, showing harmful concentration of these in the environment [45]. The combination of abiotic and biotic factors will determine the distribution and the bioaccumulation of contaminants in different tissues or organs of this species including bones, which have a high yield of collagen becoming a reliable collagen source [46,47]. Maruf et al. [48] found considerably higher concentrations of Pb and Cr in the liver of this species farmed in Bangladesh. Even Das et al. [14] found very high Cr, Pb, and Cd concentrations in tissues of *P. hypophthalmus* farmed in Bangladesh (11.03, 6.2, and 0.16 mg/kg, respectively). A consistent part of the collagen of marine origin produced came from farmed fish [49,50]. Studies on the accumulation of heavy metals in fish farms have indicated that the source of contamination could came from several factors; these include the shared one-way waste discharge system used by each pond in the farm area, the habitat conditions within the farm, the overall ecosystem health of the farm, water quality concerns within the farm system, and the potential impact of excessive use of growth supplements as feed additives [48]. Consistent differences in toxic metal concentrations were found in comparative studies between cod (*G. morhua*) and pangasius (*P. hypophthalmus*) [45].

The differences found may be attributed to various factors such as the distinct origins of these two fishes, differing methods of farming, breeding practices, feeding regimes, as well as the dimensions [51]. Additionally, variations in procedures and controls along the production chain, with more specific standards in Europe compared to Asia, could contribute to these observed differences. Nevertheless, no significant differences were found between brands of the supplements analysed for Cr, Cd, and Pb (*p*-value = 0.9349 for Cr; *p*-value = 0.6186 for Cd; *p*-value = 0.4363 for Pb). Our results on marine collagen from Tilapia spp. were significantly lower than what was found by Peng et al. [16] in Tilapia fish-scale derived collagen solution, showing Pb (1.1 mg/kg), Cr (0.91 mg/kg), and Cd (1.92 mg/kg) concentrations that exceed the regulatory standards of food industry.

The most common extraction method involves acidic solubilization using acetic acid or citric acid [8]. However, the collagen fibril of different marine organisms were not soluble in acetic acid or citric acid, even after two acidic extraction steps [52,53] thus determining a possible greater presence of toxic metals in the extract. The extraction of toxic metals from protein-rich matrices is challenging due to the interaction of metal ions with the latter.

The treatment with sulfonated polystyrene nanospheres seems to be a reliable alternative for the practical application in the sequestration of metal ions [16,54]. However, the efficiency of this treatment is strictly related to the pH of the solution. It was proven that high pH values were favourable for the removal of toxic metals. Furthermore, the decrease of collagen yield at pH = 11.5 was very limited.

Another way to prevent the presence of toxic metals during the collagen extraction can be the pre-treatment with ethylenediaminetetraacetic acid (EDTA). EDTA was proven to be the most effective agent for heavy metal decontamination [53]. Chen et al. [55] proposed a rapid method based on the hydrophilic ultrafiltration for the isolation of high purity pepsin-soluble type I collagen from the scales of *Sciaenops ocellatus.* The method was proven to be more efficient than the standard collagen extraction protocols based on salt precipitation with dialysis, showing toxic element concentrations below the Chinese national standards albeit up to 40 times higher than those found in this work for Cd, Pb, and Cr (0.0410 ± 0.0003, 0.450 ± 0.005, and 1.90 ± 0.03 mg/kg, respectively).

Even the use of the jellyfish for collagen extraction was proven to be an alternative for a product free of heavy metal contamination [8], as confirmed in this work. The highest collagen yield was achieved with *R. pulmo* oral arms (2–10 mg collagen/g of wet tissue), followed by *Cotylorhiza tuberculata* (0.45 mg/g) [56]. Furthermore, collagen from *R. pulmo* exhibits a biological impact on human cells comparable to that of mammalian type I collagen, as determined by cytotoxicity and adhesion assays [56].

Consistent with previous studies, levels of toxic metals remained below cautionary thresholds required for the potential utilization of jellyfish biomass in nutritional, nutraceutical, cosmeceutical, and pharmaceutical applications [57].

None of the samples analysed in this work exceed the maximum concentration levels imposed by the European Union [22,58]. These limits are 3 mg/kg for Cd, 3 mg/kg for Pb, and 0.1 mg/kg for Hg.

The comparison with the marine collagen samples examined by Augustsson et al. [22] show comparable Cr and Cd contents but Pb levels up to five times higher, probably due to differences related to the type of organisms used for the marine collagen production. Unfortunately, Augustsson et al. [22] did not report the origin of the marine collagen examined probably due to the lack of detailed information of the supplements collected. This problem that we also encountered forced us to reduce the sampling plan to only the brands that provided detailed information of the origin of the product. The significant variability in Pb and As content in marine collagen, coupled with the daily consumption of these supplements, stresses the need for enhanced risk assessment and quality control measures for these products. However, none of the analysed products gave ADDs > TDI for either Cd, Pb, or iAs (Table 4), in accordance to what was found by Augustsson et al. [22] in marine collagen samples.

**Table 4:** ADDs (µg/kg/day) of iAs, Cd, and Pb with available TDIs, associated with each investigated marine collagen supplement

Element (TDI value)	Brand 1	Brand 2	Brand 4	Total
iAs (0.3 µg/kg/day) [36]	4.7 × 10^−2^ (0)	5 × 10^−2^ (0)	4 × 10^−2^ (0)	4.9 × 10^−2^ (0)
Pb (0.5 µg/kg/day) [38]	9 × 10^−3^ (0)	1.9 × 10^−2^ (0)	1 × 10^−2^ (0)	1.4 × 10^−2^ (0)
Cd (0.36 µg/kg/day) [37]	2 × 10^−4^ (0)	5 × 10^−4^ (0)	2 × 10^−4^ (0)	3 × 10^−4^ (0)

Values in parentheses = ADDs in % of TDIs.

Despite the significantly higher Pb values found in this work compared to what was found by Augustsson et al. [22], the low ADD values may be related to the amount of supplement taken daily. However, health agencies have faced challenges in establishing or sustaining a reference value for Pb because of the absence of a threshold value in dose–response analyses. Despite this, numerous parameters were removed in 2011 upon the realization that there are no safe levels for Pb [59]. Pb has no beneficial function in humans. Exposure can occur through food, contaminated air, and dust, and it is transported and bound to erythrocytes, where it accumulates in different organs and tissues [60]. Pb can cause oxidative damage in various organs by directly influencing membrane lipid peroxidation and diminishing antioxidant parameters [21,61]. Studies have indicated that low levels of Pb exposure can have immunostimulant effects, whereas higher levels of exposure typically result in immunosuppression [62]. Moreover, an association between cardiovascular mortality and chronic exposure to high occupational Pb levels [63] and a correlation between Pb and beta-thalassemia patients were found [60].

## 4 Conclusions

The results of this work showed that the marine collagen supplements investigated are safe for consumers in recommended doses. Nevertheless, the supplements analysed showed alarming Pb levels probably related to the fish used for the collagen production; this could be confirmed to the extreme variability in Pb levels between brands, influenced by the product selection. However, the risk assessment carried out in this work was restricted to the toxic metals with available TDIs.

This work also does not take into account the fact that some consumers use more than one supplement per day at the same time, stressing the need for more rigorous control regarding the supplement use. Considering the results of this work and those of other researchers, it has been observed that food supplements can contain hazardous metals and metalloids at levels that substantially contribute to regular dietary intake. Moreover, the concentrations of these elements may exhibit greater variability in food supplements compared to other food types and beverages [64]. Consequently, there is a compelling need to have more data on the presence of heavy metals and metalloids to revaluate the regulations and monitoring practices for food supplements. It is suggested that producers should be compelled to analyse the content of toxic metals to prevent their occurrence in harmful concentrations in their products.
